# Priority-Based Data Collection for UAV-Aided Mobile Sensor Network

**DOI:** 10.3390/s20113034

**Published:** 2020-05-27

**Authors:** Xiaoyan Ma, Tianyi Liu, Song Liu, Rahim Kacimi, Riadh Dhaou

**Affiliations:** 1 College of Architecture and Urban Planning, Tongji University, Shanghai 200092, China; xiaoyan.ma@enseeiht.fr (X.M.); liusong5@tongji.edu.cn (S.L.); 2 School of Aerospace Engineering and Applied Mechanics, Tongji University, Shanghai 200092, China; 3 IRIT-UPS, University of Toulouse, 31062 Toulouse, France; rahim.kacimi@irit.fr; 4 IRIT-ENSEEIHT, University of Toulouse, 31071 Toulouse, France; riadh.dhaou@enseeiht.fr

**Keywords:** wireless sensor networks, multiple unmanned aerial vehicles, mobile nodes, data collection, collision-free

## Abstract

In this work, we study data collection in multiple unmanned aerial vehicle (UAV)-aided mobile wireless sensor networks (WSNs). The network topology is changing due to the mobility of the UAVs and the sensor nodes, so the design of efficient data collection protocols is a major concern. We address such high dynamic network and propose two mechanisms: prioritized-based contact-duration frame selection mechanism (PCdFS), and prioritized-based multiple contact-duration frame selection mechanisms (PMCdFS) to build collision-free scheduling and balance the nodes between the multi-UAV respectively. Based on the two mechanisms, we proposed a Balance algorithm to conduct the collision-free communication between the mobile nodes and the multi-UAVs. Two key design ideas for a Balance algorithm are: (a) no need of higher priority for those nodes that have lower transmission rate between them and the UAV and (b) improve the communication opportunity for those nodes that have shorter contact duration with the UAVs. We demonstrate the performance of proposed algorithms through extensive simulations, and real experiments. These experiments using 15 mobile nodes at a path with 10 intersections and 1 island, present that network fairness is efficiently enhanced. We also confirm the applicability of proposed algorithms in a challenging and realistic scenario through numerous experiments on a path at Tongji campus in Shanghai, China.

## 1. Introduction

Unmanned Aerial Vehicle-aided wireless sensor networks (UAV-aided WSN) have gained more and more interest due to their many applications in monitoring, surveillance, and exploring in healthcare, agriculture, industry, and military [1,2,3,4,5]. Among UAVs’ applications, one of the key functions is the data collection [6,7,8,9,10,11]. These works focus on deterministic topology where the nodes are deployed statically, and the locations of the sensors are known. The data collection issues addressed on dynamic topology, which are usually used in applications such as maritime detection, traffic surveillance, and wilderness rescuing where the targets are moving and no static sensors are deployed in advance, are seldom covered.

The main difference between the static network and mobile network are: the transmission opportunities for nodes that are within the coverage of the UAV are different. In static case, all covered nodes are static, the relative velocity ($v_{r}$) between the nodes and the UAV are the same. Thus, the contact durations (CD) between them with the UAV depend on the relative distance ($d_{r}$) between them ($CD=\frac{d_{r}}{v_{r}}$, see [12,13] for more details). The relative distances almost have no difference if the UAV flies at a higher altitude. However, in mobile case, when the nodes move at different velocities, the CD are different greatly even the relative distance is the same. Intuitively, the shorter the CD between them, the smaller the opportunities for the mobile node to communicate with the UAV. When the CD is very short, the mobile node may have no opportunity to communicate with the UAV if no attention is paid on the CD between it with the UAV. Thus, a contact-duration-based data collection algorithm should be designed for such context despite a large array of existing data collection algorithms (see Section 2. related works) on UAV-aided static WSNs.

The impact factors of the CD between mobile nodes and the UAV include two aspects: (a) the relative distance between the sensor and the UAV, and (b) the relative velocity between them. Priority-based Frame Selection (PFS) [14,15] is a one-hop mechanism based on the relative distance according to which the nodes are divided into different priority groups. Communications are conducted from higher to lower priorities. A multi-hop highest velocity opportunistic algorithm which is based on relative velocity between mobile nodes and the UAV is proposed in [16]. The ones that have higher velocity have longer CD with the UAV, therefore were selected as forwarded nodes. In our previous work [12,13], we studied the data collection maximization issues in single UAV enabled mobile WSN where the pre-defined path is a straight path without comparison with existing works and real experiments. The curve path and multi-UAVs aspects are also not covered in the previous work. Thus, a large room for enhancing the network performance still exists.

**Figure 1:**
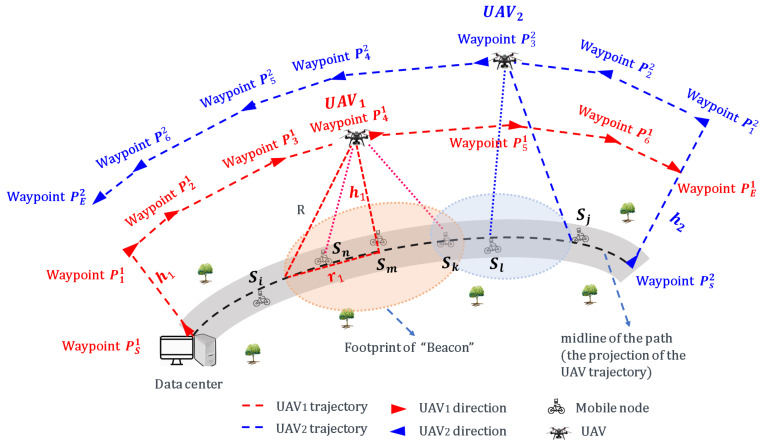
An illustration of the unmanned aerial vehicle (UAV)-aided data collection for a mobile wireless sensor network. The exemplar trajectory of the $UAV_{1}$ is shown as: Waypoint $P_{S}^{1}$→ Waypoint $P_{1}^{1}$→ Waypoint $P_{2}^{1}$→ Waypoint $P_{3}^{1}$→ Waypoint $P_{4}^{1}$→ Waypoint $P_{5}^{1}$→ Waypoint $P_{6}^{1}$→ Waypoint $P_{E}^{1}$.

In this work, we focus on multi-UAV aided mobile WSN, Figure 1, where the nodes are deployed on mobile bicycles and move along a pre-defined curve path. Considering that, in the context of the nodes move along a path, two UAVs are enough to cover all mobile nodes when (as in Figure 1) $UAV_{1}$ take-off from the original point of the path and fly along the path, $UAV_{2}$ take-off from the end-point of the path and fly along the path. Data collection issues in such contexts contain two aspects. End-to-end data collection is a very complex problem. In this paper, we focus on the access link. As the literature, on this kind of link between the sensors and the UAV [6,7], still does not propose efficient solutions. The access link suffers from the synchronization problem due to the high dynamic network, the coordination between the mobile nodes and the multi-UAVs. Providing the opportunity of communication to the nodes that have a very short duration with the UAVs reduces the congestion risk. On the other hand, extensive literature can be referred to, on the second link, on the backhaul link, between the UAVs and gateways [17]. The second link is also challenging on several levels such as the data security, the security of UAVs, and the dimensioning of the backhaul. In our previous work [18,19], we focused on the backahul link with the satellite system. The proposed algorithms on mobile mules, in [18,19] are applicable for UAV-aided sensor networks. Moreover, because that the collected data (considering the value of data and distinguish the data collected from each sensor) could be stored in SD cards embedded on the UAV, thus, in this work, we focus on the access link. The data collection optimization objectives in such context include two aspects: (i) maximizing the number of collected packets, and (ii) maximizing the number of nodes that successfully send at least one packet during the collection period. Our main purpose is to jointly maximize the two aspects through formulating the dynamic parameters. Our main contributions are summarized as follows:

- We study the impact of dynamic parameters, including the speed and flying height of UAV, the sensor speed, network size, and different priority areas. We mathematically formulate the data collection issue into the optimization with the objective of maximizing the number of collected packets and the number of sensors that successfully send packets to the UAVs.
- Based on the dynamic parameters, we adopt a time-discrete mechanism and propose a prioritized-based multiple contact-duration frame selection algorithm (PMCdFS). PMCdFS algorithm is used for the balancing between the nodes (that are within the range of multi-UAVs at the same time) and multi-UAVs.
- We improve the contact duration mechanism in our previous work (see [12,13] for more details) with the Prioritized Frame Selection (PFS) mechanism (see [14,15] for more details) and propose a prioritized-based contact-duration frame selection algorithm (PCdFS). PCdFS algorithm is a one-hop and slotted mechanism which is used to allocate the time-slot for the nodes that covered only by one of the UAVs.
- We propose a Balance algorithm to solve the collision between the nodes and UAVs so as to optimize the aforementioned data collection performance.
- Through extensive simulations, and real experiments, we examine the effectiveness of the proposed algorithms, and compare it with existing algorithm under different configurations.

The remainder of this paper is organized as follows: in the next section, we discuss previous related work. Section 3 presents the system model and the problems formulated. Section 4 present the proposed algorithms. Section 5 evaluated the proposed algorithms through extensive simulations and real experiments. Section 6 concludes this paper and gives some future work suggestions.

## 2. Related Works

There exists an extensive array of research on data collection in UAV-aided WSN with different objectives ranging from completion time minimization [20], power controlling [21], trajectory distance minimizing [22] to energy consumption minimization [23,24]. We classify these existing data collection algorithms by two criteria: (i) Static or mobile nodes, and (ii) sensors are deployed along a path or deployed within an interesting area. In (i), algorithms are differentiated by whether the sensors mobile or not because the dynamic parameters brought by the movement of nodes in the network structure have a much greater impact on the system performance. In (ii), algorithms are differentiated by whether the nodes deployed along a path or not. The nodes deployed along a given path [12,13,25,26] so the UAV trajectory planning has very little impact on the network performance.

(i) Data collection algorithms addressed on mobile nodes. There are many works on studying how to collect data from WSN. The authors in [4,9,27,28,29] review these works. According to the [4,9,27,28,29], most of these algorithms only based on the mobile sink or only focused on mobile sensors. In our previous works [12,13,16], we studied how to use UAV to collect data from mobile nodes based on an assumption that both the nodes and the UAV move along a straight path with constant speeds. The case where both the UAV and the nodes move in a curved path is not considered. Numerous researches have been done on statically deployed networks [6,7,11,14,15,20,24,25,30,31,32,33,34,35,36,37,38,39,40].

(ii) Most of the aforementioned data collection algorithms can also be classified according to the deployed status of the nodes. Authors in [12,13,16,25,34] studied how to use UAV to collect data from nodes that deployed along a straight path. Especially in [25], the nodes deployed on a straight line, and the UAV flies over this line to collect data from nodes. In such context, the trajectory of the UAV is dependent on the path (or line) and has a light impact on the performance if the path is long enough. For instance, in [25], the authors aim to minimize the flight time through jointly optimizing the transmit power of nodes, the UAV speed and the transmission intervals. For the case that nodes are deployed within the area of interest, one of the main issues is to plan the UAV’s trajectory so as to enhance the network performance. Numerous research has been done on the UAV trajectory planning issues [6,7,20,24,30,31,32,33,34,35,36,37,38,39,40]. These works are different from the optimization method and objective function because of different scenarios. They are mainly classified into two types: single-UAV trajectory planning [6,7,24,30,31,32,33,34] and multi-UAV trajectory planning [20,35,36,37,38,39,40].

The first is the single-UAV trajectory planning. Authors in [33] use a UAV for the mobile edge computing system. They minimize the maximum delay of all ground users through jointly optimizing the offloading ratio, the users’ scheduling variables, and UAV’s trajectory. While, in [24], the authors aim to minimize the maximum energy consumption by optimizing the trajectory of a rotary-wing UAV. The authors utilize a UAV to collect data from IoT devices with each has limited buffer size and target data upload deadline [6]. In this study, the data should be transmitted before it loses its meaning or becomes irrelevant. To maximize the number of served IoT devices, they jointly optimize the radio resource allocation and the UAV’s trajectory.

The second is the multi-UAV trajectory planning. Multi-UAVs were used as mobile base stations to provide service for ground users in [38]. They aim to maximize the minimum throughput of ground users by optimizing the trajectory for each UAV. Scholars in [20] employ multi-UAVs to collect data from nodes. Through jointly optimizing the trajectories of UAVs, wake-up association and scheduling for sensors, they minimize the maximum mission completion time of all UAVs. The authors studied a multiple casting network utilizing the UAV to send files to all ground users [37]. They aim to minimize the mission completion time of the UAVs through designing the UAV’s trajectory. Meanwhile, the proposed algorithms guarantee that each ground user can successfully recover the file. In urban applications, the authors proposed a risk-aware trajectory planning algorithm [36] for multi-UAVs. Under the same test scenarios, authors in [39] aim to minimize the mission time by planning the trajectory of each UAV. The scholars exploit the nested Markov chains to analyze the probability for successful data transmission [40]. They propose a sense-and-send mechanism [40] for real-time sensing missions, and a multi-UAVs enabled Q-learning algorithm for decentralized UAV trajectory planning.

**Figure 2:**
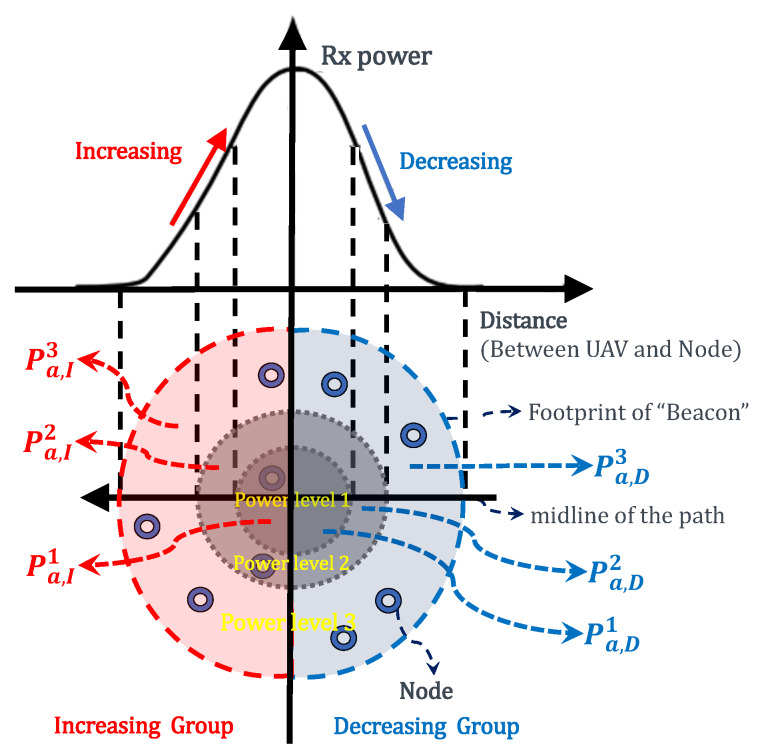
The Priority Frame Selection (PFS) mechanism.

In other cases. The authors in [11] use a single UAV to collect data from harsh terrains. Due to the large scale of the detection area, the network has a high demand for power. They adopted a rechargeable mechanism to extend the lifetime of the UAV so as to enhance the collection period. The PFS mechanism in [14,15] is based on the nodes’ positions for the data collection in single-UAV aided static sensor networks. The nodes are divided into different priority groups according to two steps: (i). increasing group and decreasing group (Figure 2). The nodes within the decreasing group was given higher priority than the ones within the increasing group. (ii). For each group in (i), the nodes were divided into sub-groups according to which power level does it belong to. The sets of nodes within “power level 1” in the increasing group and in the decreasing group are denoted by $S_{a,I}^{1}$ and $S_{a,D}^{1}$, respectively. The priority values for nodes within $S_{a,I}^{1}$ and $S_{a,D}^{1}$ are denoted by $P_{a,I}^{1}$ and $P_{a,D}^{1}$, respectively. The authors give high priority to those nodes that are within high power level (Figure 2), and applied opposite actions to the increasing and decreasing groups: (a) in the increasing group, the nodes within high power level was given high priority value; (b) in the decreasing group, the nodes within lower power level were given high priority. After these actions, almost all nodes at the best channel conditions have been considered.

Table 1 presents the key focuses and the key difference of our proposed algorithms from existing algorithms. Although a lot of research has been done on data collection, there is still room to enhance the network performance through balancing the dynamic parameters in the first link in mobile sensor networks.

## 3. System Model and Problem Formulation

### 3.1. System Model

This paper considers a UAV-assisted mobile sensor network which has *N* mobile bicycles with each equipped a sensor, and *M* UAVs with each equipped a sensor (as illustrated in Figure 1, where *M* = 2). $S=\{S_{1},S_{2},\cdots ,S_{N}\}$ is the set of mobile sensors. *N* nodes move along a pre-defined path (path length is denoted as *L*) with each has a speed $v_{i}$. The UAV $U_{i}$ is dispatched to collect data from mobile sensors at a given height $h_{i}$ and speed $v_{u}^{i}$ along a predefined trajectory (Figure 1).

The trajectory consists of a few line segments that contain the waypoint start and waypoint end (e.g., in Figure 1, waypoint $P_{S}^{i}$ and waypoint $P_{E}^{i}$ in the trajectory of $UAV_{i}$, *i* = 1, 2), and *k* intermediate waypoints (e.g., in Figure 1, waypoint $P_{1}^{i}$, waypoint $P_{2}^{i}$, waypoint $P_{3}^{i}$, waypoint $P_{4}^{i}$, waypoint $P_{5}^{i}$ and waypoint $P_{6}^{i}$ in the trajectory of $UAV_{i}$, *i* = 1, 2). Let $P_{i}=\left\{P_{S}^{i},P_{1}^{i},P_{2}^{i},\cdots ,P_{k}^{i},P_{E}^{i}\right\}$ denote the set of all waypoints of $UAV_{i}$. The coordinates for each waypoint $P_{m}^{i}$ is denoted by $P_{m}^{i}\left(x_{m}^{i},y_{m}^{i},h_{i}\right)$. The UAV’s flight time between any two waypoints $P_{m}^{i}$ and $P_{n}^{i}$ is given by,

(1) $$\lambda_{m,n}^{i}=\frac{\|P_{m}^{i}-P_{n}^{i}\|}{v_{u}^{i}}\;,\;P_{m}^{i},P_{n}^{i}\in P_{i}.$$

The collection period of the $UAV_{i}$ is the duration from waypoint $P_{1}^{i}$ to the waypoint $P_{E}^{i}$. It is denoted by $T_{i}$,

(2) $$T_{i}=\sum_{m=1}^{k-1}\lambda_{m,m+1}^{i}+\lambda_{k,E}^{i}.$$

The trajectory length for $UAV_{i}$ is,

(3) $$L_{i}=\sum_{m=1}^{k-1}\|P_{m+1}^{i}-P_{m}^{i}\left\|\;+\;\right\|P_{E}^{i}-P_{k}^{i}\|.$$

Generally, in a given path, the coordinates (x-axis and y-axis) of the waypoints for the UAVs are the same except the height (z-axis). For instance, the point $\left(x_{m}^{i},y_{m}^{i},h_{j}\right)$ is one of the waypoints for $UAV_{j}$ (i.e., $P_{m}^{j}\left(x_{m}^{i},y_{m}^{i},h_{j}\right)\in P_{j}$) if $P_{m}^{i}\left(x_{m}^{i},y_{m}^{i},h_{i}\right)\in P_{i}$. Thus, we have $L_{i}=L_{j}$. Intuitively, the straighter the pre-defined path, the smaller the ΔL ($\Delta L=|L\;-\;L_{i}|$). The larger the number of waypoints, the smaller the ΔL. Major notations used in this work are defined in Table 2.

To well present the impact of the dynamic parameters on the system, we using homogeneous UAVs ($v_{u}^{i}=v$) to reduce the influence brought by UAVs’ speeds. Accordingly, the collecting period is denoted by *T*, and $T=T_{i}$.

### 3.2. Discrete Time Mechanism

**Figure 3:**
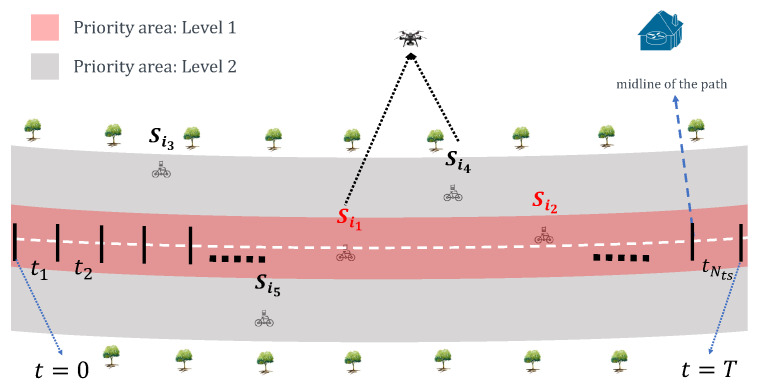
An illustration of studied scenario.

Considering the waypoint selection and beacon sending, we introduce a discrete-time mechanism where the collecting period *T* is divided into $N_{ts}$ time-slots with each lasting α time units, $N_{ts}=\left\lfloor \frac{T}{\alpha }\right\rfloor$, where ⌊·⌋ is the rounding down function. It is assumed that the time-slots are indexed as $1,2,\cdots ,N_{ts}$, and $T=\{t_{1},t_{2},\cdots ,t_{N_{ts}}\}$ (Figure 3). It is worth note that, in each time slot, a sensor could communicate only with one UAV. For example, in $t_{k}$, $S_{i}$ communicate with $UAV_{m}$, and $S_{j}$ communicate with $UAV_{n}$ (i≠j and m≠n).

From Figure 1, the nodes that are covered by the $UAV_{i}$ and deployed nearly complete to communicate with the $UAV_{i}$. For instance, $S_{m},S_{n},S_{k}$ in Figure 1 complete to communicate with the $UAV_{1}$. Meanwhile, there are more than one UAV within the range of one node. For example, $S_{k}$ in Figure 1 with the range of both $UAV_{1}$ and $UAV_{2}$. The $S_{k}$ should choose one from them to send packets. Hence, how to balance the communication between nodes and the UAVs so as to maximize the data collection is a challenging task.

### 3.3. Data Collection Protocols Using UAV

**Figure 4:**
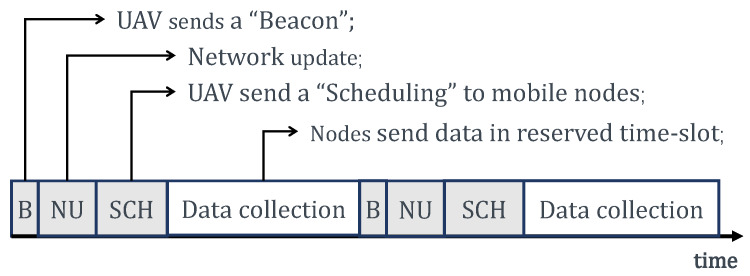
The procedure of allocating.

In this paper, we present a distributed method for the data collection issues in UAV-aided mobile sensor networks as follows. The collection period *T* is divided into $N_{ts}$ time slots. At the beginning of every time slot (Figure 4), UAV sends a beacon message to tell the mobile nodes that UAV is coming. The beacon includes the UAV’s information, e.g., the aerial height, speed, etc. The new comers send a JOIN message which includes the sensors’ information to the UAV to update the network topology. The UAV judges whether the nodes are within its range or not according to these messages. Then, it calculates the contact duration, the relative distance, and the potential time slots for each node that successfully sends the JOIN message. According to the time slot allocation algorithms that we proposed in Section 4, the UAV provides scheduling for the covered sensors, and broadcasts them a scheduling message which contains the assignment of the time-slots. Having received the scheduling message, every sensor transmits its data in its own time slots.

#### 3.3.1. Collecting Packets

Allocating the $N_{ts}$ time slots to individual mobile sensors under the proposed mechanism is equivalent to maximizing the usage of time slots. Let

$$N_{ts,a}\left(i,j,k\right)=\left\{\begin{array}{cc} 1 & UAV_{i}communicatewithS_{j}int_{k}\;,\\ 0 & otherwise. \end{array}\right.$$

The data collection maximization problem is to maximize the number of collected packets, $N_{p}$,

(4) $$N_{p}=\sum_{i=1}^{M}\sum_{j=1}^{N}\sum_{k=1}^{N_{ts}}N_{ts,a}\left(i,j,k\right)\cdot \sigma_{ijk}\cdot D_{r}\cdot \alpha \;.$$

where $D_{r}$ is the transmission rate, and

$$\sigma_{ijk}=\left\{\begin{array}{cc} 1 & successfullytransmission\;,\\ 0 & otherwise. \end{array}\right.$$

Our objective is to balance the communication between the two UAVs and *N* mobile nodes to maximize the overall data collection utility. Therefore, the optimization problem can be formulated as,    

(5) $$\begin{array}{ccc} P_{1} & : & \max_{S_{j}\in S,t_{k}\in T}\;\left\{N_{p}\right\}\;, \end{array}$$

(6) $$\begin{array}{cc} s.t. & \sum_{k=1}^{N_{ts}}N_{ts,a}\left(i,j,k\right)\leq N_{ts}\;,\forall i,j\;, \end{array}$$

(7) $$\begin{array}{c} \sum_{j=1}^{N}N_{ts,a}\left(i,j,k\right)\leq N\;,\forall i,k\;, \end{array}$$

(8) $$\begin{array}{c} \sum_{i=1}^{M}N_{ts,a}\left(i,j,k\right)\leq M\;,\forall j,k\;, \end{array}$$

(9) $$\begin{array}{c} \sum_{i=1}^{M}\sum_{j=1}^{N}\sum_{k=1}^{N_{ts}}N_{ts,a}\left(i,j,k\right)\leq M\cdot N_{ts}\;,\forall i,j\;. \end{array}$$

Constraints (6)–(8) imply that, in a given time-slot, a UAV chooses only one node to collect data, and one node selects only one UAV to send data. Constraint (9) ensures that, in a given time-slot, no more than two communications happen between UAVs and mobile nodes.

#### 3.3.2. The Number of Nodes that Successfully Send Packets to the UAV

During the communication between the UAVs and mobile nodes, the sensors transmission state contains: have no opportunity to send packets, have an opportunity to send but fail to transmit, and successfully send data to the UAVs. The larger number of nodes ($N_{node}$) that successfully transmit packets, the higher the system performance. Thus, to enhance the number of nodes that successfully send data to the UAVs is one of the key points in designing data collection algorithms.

Let matrix $I_{M\times N\times N_{ts}}$ is given by,

$$I_{ijk}=N_{ts,a}\left(i,j,k\right)\cdot \sigma_{ijk}\cdot i\;,\;\;UAV_{i}\in U,S_{j}\in Sandt_{k}\in T.$$

The elements in matrix *I* are the node ID. Then, we can obtain the number of nodes that successfully transmit at least one packet,

(10) $$N_{node}≜Hist\left(I\right).$$

where “Hist” is used to calculated the number of different elements in the *I* matrix. The $N_{node}$ maximization problem can be regarded as the formulated problem,

(11) $$P_{2}:\max_{S_{j}\in S,t_{k}\in T}\;\left\{N_{node}\right\}\;,$$

(12) $$\begin{array}{cc} s.t. & \sum_{k=1}^{N_{ts}}N_{ts,a}\left(i,j,k\right)\leq N_{ts}\;,\forall i,j\;, \end{array}$$

(13) $$\begin{array}{c} \sum_{j=1}^{N}N_{ts,a}\left(i,j,k\right)\leq N\;,\forall i,k\;, \end{array}$$

(14) $$\begin{array}{c} \sum_{i=1}^{M}N_{ts,a}\left(i,j,k\right)\leq M\;,\forall j,k\;, \end{array}$$

(15) $$\begin{array}{c} \sum_{i=1}^{M}\sum_{j=1}^{N}\sum_{k=1}^{N_{ts}}N_{ts,a}\left(i,j,k\right)\leq M\cdot N_{ts}\;,\forall i,j\;. \end{array}$$

When *i* = 1 (single-UAV enabled sensor network), it is a classical NP-hard problem that we have studied in [12,13]. When *i* = 2 (multi-UAV enabled sensor network), this problem is also an NP-hard combinatorial maximization problem [41]: under the given conditions, its objective is to select items which have unique weight and value to maximize the total value.

## 4. Proposed Algorithms

In this section, we study how to balance the communication between multi-UAVs and mobile nodes, and we propose a balance mechanism. For the two cases, multiple nodes within the range of both two UAVs and multiple nodes only with the range of only one UAV, we propose two algorithms: PCdFS (Section 4.2) and PMCdFS (Section 4.3) algorithms.

### 4.1. Balance Algorithm between UAVs and Mobile Nodes

In a given time slot $t_{k}$ ($t_{k}\in$ T), there are multiple nodes within the range of the UAV. The nodes that are potentially for $UAV_{1}$ and $UAV_{2}$ are denoted by $S_{kB}^{1}$ and $S_{kB}^{2}$ respectively. When $S_{kB}^{1}\cap S_{kB}^{2}$ = ⌀, there is no node within the range of the $UAV_{1}$ and $UAV_{2}$ at the same time. In this case, we propose PCdFS mechanism (see Section 4.2 for more details) to balance the communications between $S_{kB}^{1}$ and $UAV_{1}$, $S_{kB}^{2}$ and $UAV_{2}$ respectively. When $S_{kB}^{1}\cap S_{kB}^{2}\neq ⌀$, and $S_{kB}^{1,2}≜S_{kB}^{1}\cap S_{kB}^{2}$. Then,

(16) $$S_{kB}^{1,o}≜S_{kB}^{1}-S_{kB}^{1,2}\;,$$

(17) $$S_{kB}^{2,o}≜S_{kB}^{2}-S_{kB}^{1,2}\;,$$

$S_{kB}^{1,o}$ and $S_{kB}^{2,o}$ denote the sensors set only within the range of the $UAV_{1}$ and $UAV_{2}$ respectively. We use the PCdFS mechanism to balance the communications between $S_{kB}^{1,o}$ and $UAV_{1}$, $S_{kB}^{2,o}$ and $UAV_{2}$ respectively. For the nodes within $S_{kB}^{1,2}$, we proposed the PMCdFS algorithm to balance between $|S_{kB}^{1,2}|$ mobile nodes and multi-UAVs. The Balance algorithm is detailed in Algorithm 1.
**Algorithm 1** Balance Algorithm.**Input:** Initial deployed information of nodes and UAVs**Output:***$N_{p}$, $N_{node}$*  1: $N_{p}$ = $N_{node}$ = 0, *k* = 1, $T_{now}$ = 0;  2: **Step 1. Synchronization;**  3: UAV sends *k*-th ’Beacon’ message;  4: Network update, obtain the $S_{k_{B}}^{1}$ and $S_{k_{B}}^{2}$;  5: **Step 2. Data Communication;**  6: **while**
$T_{now}<T$
**do**  7:        Let $S_{kB}^{1,2}≜S_{kB}^{1}\cap S_{kB}^{2}$, $S_{kB}^{1,o}≜S_{kB}^{1}-S_{kB}^{1,2}$, and $S_{kB}^{2,o}≜S_{kB}^{2}-S_{kB}^{1,2}$;  8:        **if**
$S_{kB}^{1,2}$ = ⌀ **then**  9:                Apply PCdFS mechanism (Algorithm 2) to balance the communication between $S_{kB}^{1,o}$ and $UAV_{1}$, $S_{kB}^{2,o}$ and $UAV_{2}$ respectively;10:        **else**11:                Apply PMCdFS algorithm (Algorithm 3) for the balancing between mobile nodes in $S_{kB}^{1,2}$ and multi-UAVs, and obtain $S_{k_{B}}^{1}$ and $S_{k_{B}}^{2}$ through PMCdFS algorithm;12:                Apply PCdFS mechanism (Algorithm 2) to balance the communication between $S_{kB}^{1,o}$ and $UAV_{1}$, $S_{kB}^{2,o}$ and $UAV_{2}$ respectively;13:        **end if**14:        Update $T_{now}$, *k*, $N_{p}$ and $N_{node}$;15: **end while**16: **return**
$N_{p}$ and $N_{node}$;

### 4.2. Priority-Based Contact-Duration Frame Selection Mechanism

**Figure 5:**
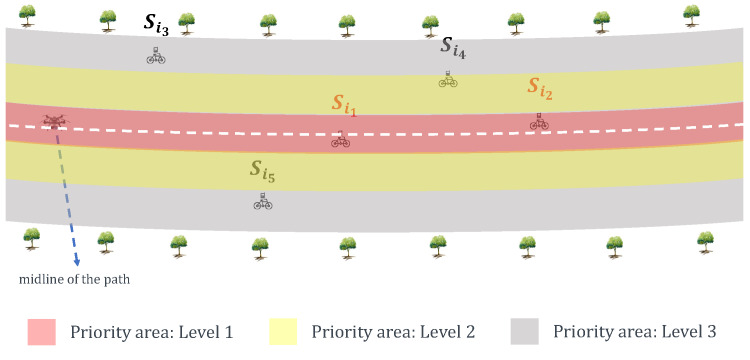
Priority areas.

In the PCdFS mechanism, the priority areas division includes two steps: (i) divide the nodes into different groups according to their power level. For example, the nodes are divided into two groups and three groups in Figure 3 and Figure 5, respectively. In Figure 3, $S_{i_{1}}$ and $S_{i_{2}}$ are within the same priority area (level 2), $S_{i_{3}}$, $S_{i_{4}}$ and $S_{i_{5}}$ are within level 2. If we take more priority levels into account, e.g., 3 levels as in Figure 5, $S_{i_{1}}$ and $S_{i_{2}}$ belong to level 1, $S_{i_{4}}$ is in level 2, $S_{i_{3}}$ and $S_{i_{5}}$ are in level 3. The more levels, the more detailed group. (ii) For each group, the nodes are given different priority according to their contact duration with the UAV. The ones that have short CD with the UAV are given higher priority values. In PCdFS, different nodes are given different priority values except the case that more than one node have the same CD with the UAV. In PCdFS, it makes the nodes facing a connection lose with the UAV highly concerned. In addition, PCdFS provides the nodes within a higher power level to send data exactly at the moment of their good channel condition so as to reduce the packet’s loss. The PCdFS algorithm is detailed in Algorithm 2.
**Algorithm 2** Prioritized-based contact-duration frame selection mechanism (PCdFS) Algorithm.**Input:** Initial deployed information of nodes and UAVs, $S_{kB}^{1}$, $S_{kB}^{2}$, $N_{p}$, $N_{node}$.**Output:***$N_{p}$, $N_{node}$*  1: **for**
$\forall S_{i}\in S_{k_{B}}^{1}$, $\forall S_{j}\in S_{k_{B}}^{2}$
**do**  2:        Make a judgement for sensor $S_{i}$ and $S_{j}$: which priority area does them in;  3:        Calculate the *contact duration* between $S_{i}$ and the $UAV_{1}$, $S_{j}$ and the $UAV_{2}$, respectively;  4: **end for**  5: For $UAV_{1}$ (and $UAV_{2}$), $t_{k}$ allocated to the one (e.g., $S_{i_{k}}$, and $S_{i_{k}}\in S_{k_{B}}^{1}$ for $UAV_{1}$, and $S_{j_{l}}$, $S_{j_{l}}\in S_{k_{B}}^{2}$ for $UAV_{2}$) which is within the higher priority area; When more than one node within the same high priority area, $t_{k}$ allocated to the one (e.g., $S_{i_{k}}$ for $UAV_{1}$, and $S_{j_{l}}$ for $UAV_{2}$) which has the shorter contact duration with the UAV.  6: In $t_{k}$, $S_{i_{k}}$ and $S_{j_{l}}$ send packets to $UAV_{1}$ and $UAV_{2}$ respectively;  7: Update $N_{p}$, $N_{node}$;  8: **return**
$N_{p}$ and $N_{node}$;

### 4.3. Priority-Based Multiple-Contact-Duration Frame Selection Mechanism

The PMCdFS algorithm is used to balance the communications between the UAVs and nodes when these nodes are within the range of the multi-UAVs at the same time. Intuitively, the longer the CD between the nodes and the UAV, the higher the opportunity to send packets to the UAV. Thus, it increases the transmission opportunity of the node if it was arranged to the UAV which has a longer CD between it and the UAV. The PMCdFS is detailed in Algorithm 3. Through the PMCdFS algorithm, we obtain the sensors set in which all nodes only compete to communicate with a single UAV ($UAV_{1}$ or $UAV_{2}$). Then, we apply the PCdFS algorithm to conduct the communication among them. The proposed algorithms are summarized in Table 3.
**Algorithm 3** Prioritized-based multiple contact-duration frame selection mechanisms (PMCdFS) algorithm.**Input:** Initial deployed information of nodes and UAVs, $S_{kB}^{1,2}$, $S_{kB}^{1}$, $S_{kB}^{2}$.**Output:**$S_{kB}^{1}$ and $S_{kB}^{2}$  1: **for**
$\forall S_{i}\in S_{k_{B}}^{1,2}$
**do**  2:        Calculate the *contact duration* between $S_{i}$ and the $UAV_{1}$ (denoted as $T_{i,1}$), the $UAV_{2}$ (denoted as $T_{i,2}$), respectively;  3:        **if**
$T_{i,1}<T_{i,2}$
**then**  4:                $S_{kB}^{2}$ = $S_{kB}^{2}\cup \left\{S_{i}\right\}$ ;  5:        **else**  6:                $S_{kB}^{1}$ = $S_{kB}^{1}\cup \left\{S_{i}\right\}$ ;  7:        **end if**  8: **end for**  9: **return**
$S_{kB}^{1}$ and $S_{kB}^{2}$;

In the following, we will evaluate the proposed algorithms through different configurations, and compare our proposed algorithms with the existing algorithm (PFS).

## 5. Implementation and Evaluation

We implement the algorithms in both simulations and real experiments as following.

### 5.1. Simulations

We conduct the simulations in MATLAB/Simulink where the UAV fly (5 min) along a path (the path is 10 m wide). The simulated priority groups are {2, 3, 4, 5} groups. The other simulation parameters are presented in Table 4, the final results are given by the mean of 30 simulation runs. Considering that, the PFS mechanism is proposed and examined based on a single-UAV sensor network. To compare it to the proposed algorithm, we use M=1 in the simulations in Section 5.1.1, Section 5.1.2, Section 5.1.3 and Section 5.1.4. In Section 5.1.5, we compare our proposed algorithms when using single UAV and multiple UAVs. All the simulations are summarized in Table 5.

#### 5.1.1. Impact of Priority Level Changes

**Figure 6:**
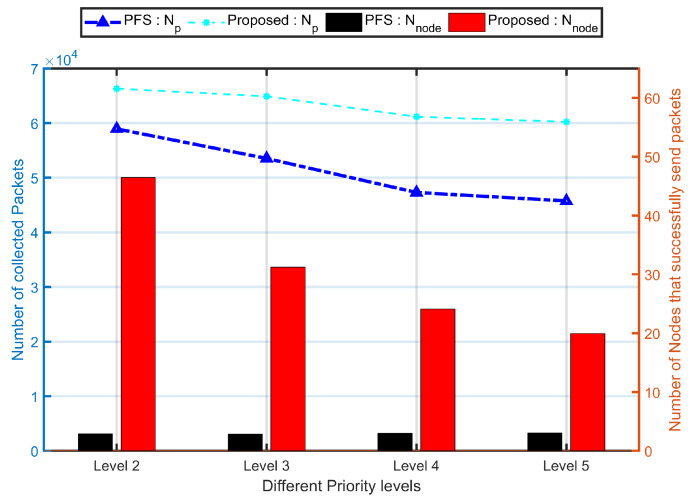
Impact of priority area change. In these simulations, the proposed algorithm is the combination of proposed Balance and prioritized-based contact-duration frame selection mechanism (PCdFS) algorithms.

Figure 6 presents the impact of varying the number of priority groups. The more priority groups, the smaller number of collected packets. The number of collected packets is much improved at two priority groups division as compared to five priority groups division. That is because the nodes in lower priority groups may have changed their state when it was their turn to send packets. The introduction of contact duration provides high priority to them so as to overcome a part of this issue, thus more packets were collected in PCdFS algorithm.

It also can be concluded that at a larger number of priority groups, a smaller number of nodes were within the highest priority group. Then, the smaller number of nodes have opportunities to send packets, which is unfair for the network. The number of nodes that successfully sent at least one packet in the proposed Priority-based Contact-duration Frame Selection mechanism was 16.2 times larger than in the PFS mechanism which is because the dynamic parameters are concerned in the proposed algorithm.

In the following, in both simulations and real experiments, the number of priority groups is fixed at 2.

#### 5.1.2. Varying Beacon Intervals

**Figure 7:**
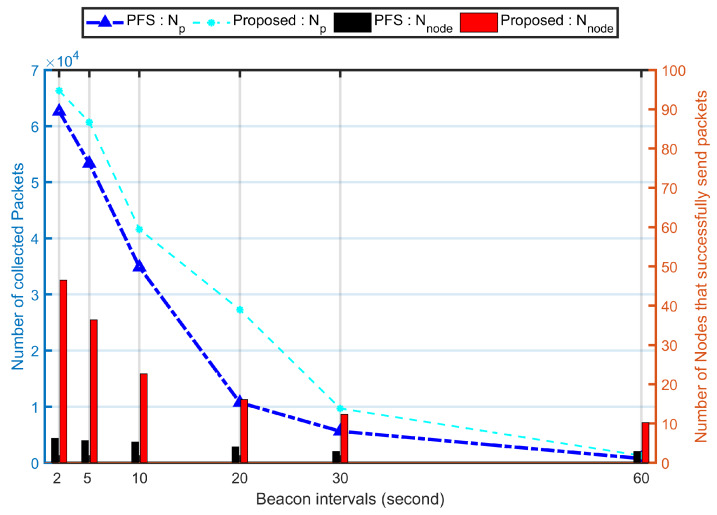
The impact of inter-beacon duration on network performance. In these simulations, the proposed algorithm is the combination of proposed Balance and PCdFS algorithms.

Figure 7 shows that both $N_{p}$ and $N_{node}$ were much improved when the inter-beacon duration at 2 s. Indeed, the longer the beacon intervals, the smaller the number of beacons sent. Thus, the number of network synchronizations is reduced so that nodes were seldom detected during collecting. No node will be detected if no beacon is sent.

#### 5.1.3. Impact of UAV’s Parameters Changes

**Figure 8:**
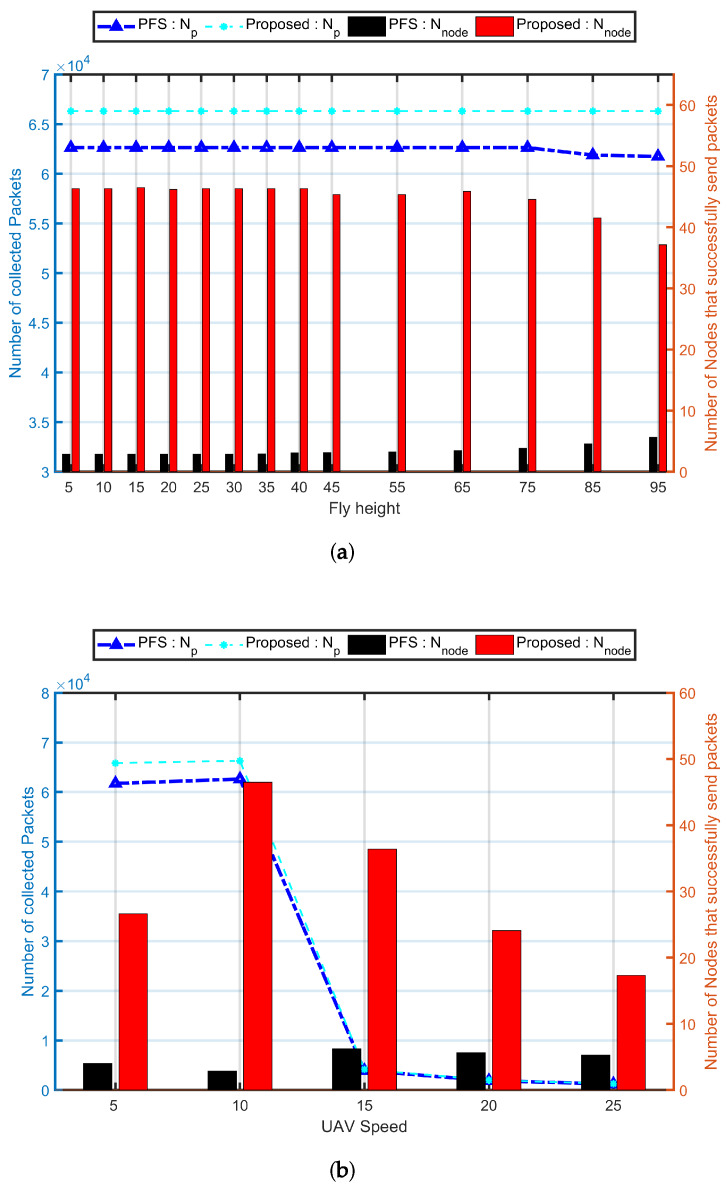
Network performance for varying UAV’ parameters: flight height and speed. In these simulations, the proposed algorithm is the combination of proposed Balance and PCdFS algorithms. (**a**) The number of collected packets for the network for varying fly height of the UAV. The number of nodes that successfully send packet to the UAV in the same scenario. (**b**) The number of collected packets for the network for varying UAV’ speed. The number of nodes that successfully send packet to the UAV for varying the speed of the UAV.

Figure 8 shows the impact of the total number of collected packets for varying the UAV speed and fly height. The network achieves the optimal ($N_{node}=46.5$ of 30 simulations) when the fly height is 15 m (Figure 8a). In this simulation, the UAV speed is 10 ms^−1^, and the size is 200 with nodes speeds vary from 1 ms^−1^ to 10 ms^−1^. Due to using fixed Dr, the flight height had very slight impact on both $N_{p}$ and $N_{node}$. The contact duration which was given by the relative distance between the nodes and the UAV was highly affected by the fly height. Hence, the PCdFS algorithm presents a difference from the PFS mechanism when the fly height is 95 m. Compared to $N_{p}$, the $N_{node}$ was affected much when the fly height is larger than 75 m. There is clearly a difference between the two mechanisms when the gap between different fly heights exceeds 50 m.

The change of the UAV speed has a huge impact on both the total number of collected packets and the number of nodes that successfully send packets to the UAV Figure 8b. When the gap between the UAV speed and the maxi speed of all nodes is very small, the network performance is optimal. In this studied scenario, the maxi speed for all nodes is 10 ms^−1^, thus, the performance is optimal when the UAV speed is 10 ms^−1^. When $V_{uav}>10$ ms^−1^, the higher the $V_{uav}$, the bigger gap between the UAV speed and the nodes’ speeds, the shorter contact duration between them, then, the less opportunities for nodes to communicate with the UAV. Then, the smaller number of packets sent to the UAV, the more it was unfair for the network.

#### 5.1.4. Scalability

**Figure 9:**
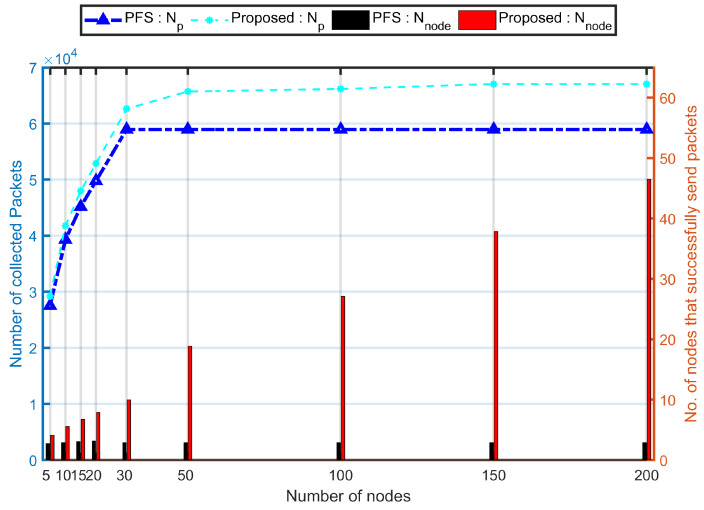
Evaluation of proposed algorithm (the combination of proposed Balance and PCdFS algorithms) on network size.

Figure 9 shows the impact of the network size on system performance. In this study, the flight height is 15 m and UAV’s speed is 10 ms^−1^ and the size vary from 5 to 200 with nodes’ speeds vary from 1 ms^−1^ to 10 ms^−1^.

The larger the network size, the larger number of nodes has the opportunity to communicate with the UAV, thus, the larger number of packets were sent to the UAV. When the size was larger than 30, each time-slot has successful communication, thus, the number of collected packets in the PFS mechanism keeps steady. It keeps increasing in the PCdFS algorithm until it reaches the transmission upper bound of the collection time. The $N_{node}$ increased steadily in the proposed algorithm. The $N_{node}$ when N=200 in the proposed algorithm is 11.34 times larger than when N=5 while it is almost the same in the PFS mechanism. Hence, the proposed algorithm shows high scalability in terms of sensors density.

#### 5.1.5. Comparison between Multi-UAVs and Single-UAV

**Figure 10:**
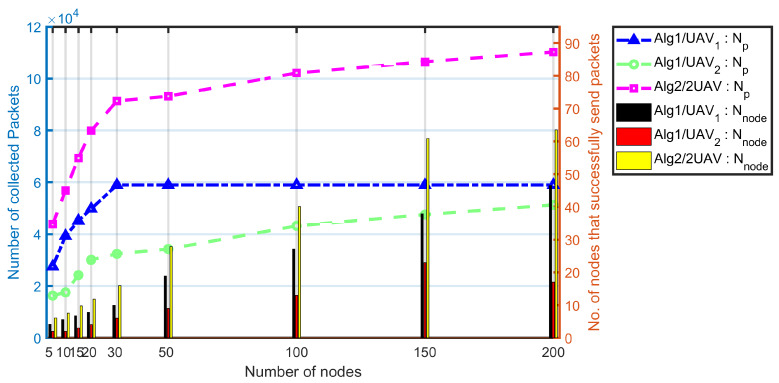
The impact of $UAV_{1}$, $UAV_{2}$ and multi-UAVs of proposed algorithm on network size. In these simulations, “Alg1” is the combination of proposed Balance and PCdFS algorithms, “Alg2” is the combination of proposed prioritized-based multiple contact-duration frame selection mechanisms (PMCdFS), PCdFS, and Balance algorithms.

Figure 10 presents the impact of proposed algorithms on the network size. “Alg1/$UAV_{1}$” simulate the combination of proposed Balance and PCdFS algorithms on the $UAV_{1}$ which takes-off from the original point of the path, while “Alg1/$UAV_{2}$” simulate the same combination algorithms on the $UAV_{2}$ which takes-off from the endpoint (the midline of the path) of the path. $UAV_{1}$ fly in the same direction as the nodes while $UAV_{2}$ fly in the opposite direction. Intuitively, the average contact duration between the $UAV_{1}$ and the nodes is longer than the average value between $UAV_{2}$ and the nodes. Thus, the communication conducted in the $UAV_{1}$ case works better than in $UAV_{2}$. There is no doubt that the multi-UAVs work better than single UAVs in data collection issues because of more opportunity provided for mobile nodes.

### 5.2. Real Experiment

#### 5.2.1. Set Up

**Figure 11:**
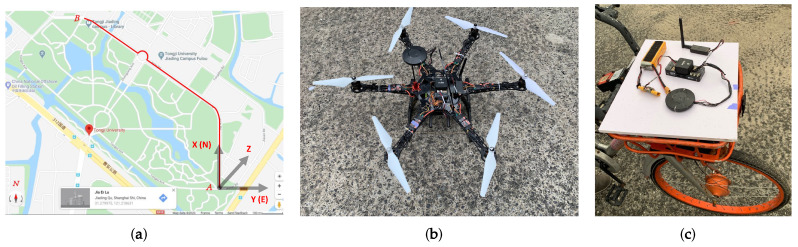
Presentation of the studied path and hardware in experiments. (**a**) Experiments path in Tongji University-Jiading Campus. (**b**) The UAV employed with a Pixhawk autopilot system. (**c**) The Pixhawk autopilot system deployed on a bicycle.

**Figure 12:**
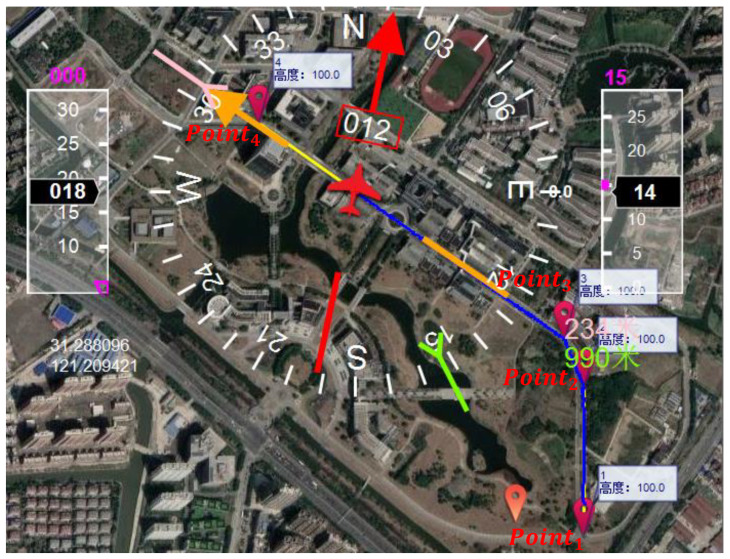
A screen shot from ground control station.

We study a path in Tongji University (Jiading Campus) as in Figure 11a. It is 5 meters wide and 1200 m long, with several intersections and 1 island (Figure 11a). In these experiments, the UAV equips a Pixhawk autopilot system [42,43] (as shown in Figure 11b) so as to fly along a predefined path at a given height. The UAV controlled through a ground station (Figure 12) where the flight height, speed and the packet transmission are controlled. We implement 15 bicycles move along the path with each equips a Pixhawk to simulate the communications based on proposed algorithms (Figure 11c). These nodes start with a random distance from the original point (point A in Figure 11a). Their locations and speeds are expressed in the NED coordinate system, as presented in Figure 11a.

Pixhawk has built-in MAVLINK protocol [44], the protocol No.24 (GPS_RAW_INT) [44] is used as the “beacon” packet (including the speed and location of the UAV) for the UAV, whose interval can be configured (e.g., in the following experiments, the beacon intervals is set at 2 s). For mobile nodes, the protocol No.24 (GPS_RAW_INT) is used as the “update” packet (including the speed and location of the mobile node). We modified and reused the protocol No.36 (SERVO_OUTPUT_RAW) [44] as the “scheduling” packet (which stores the sensor ID and time-slot ID for the collision-free communication between nodes and the UAV) for the UAV. Each MAVLINK packet contains a system ID field so we can use it to identify the sender. The pixhawk also has a log system so the GPS information, as well as the received packet number and time, is stored in the on-board SD card.

**Figure 13:**
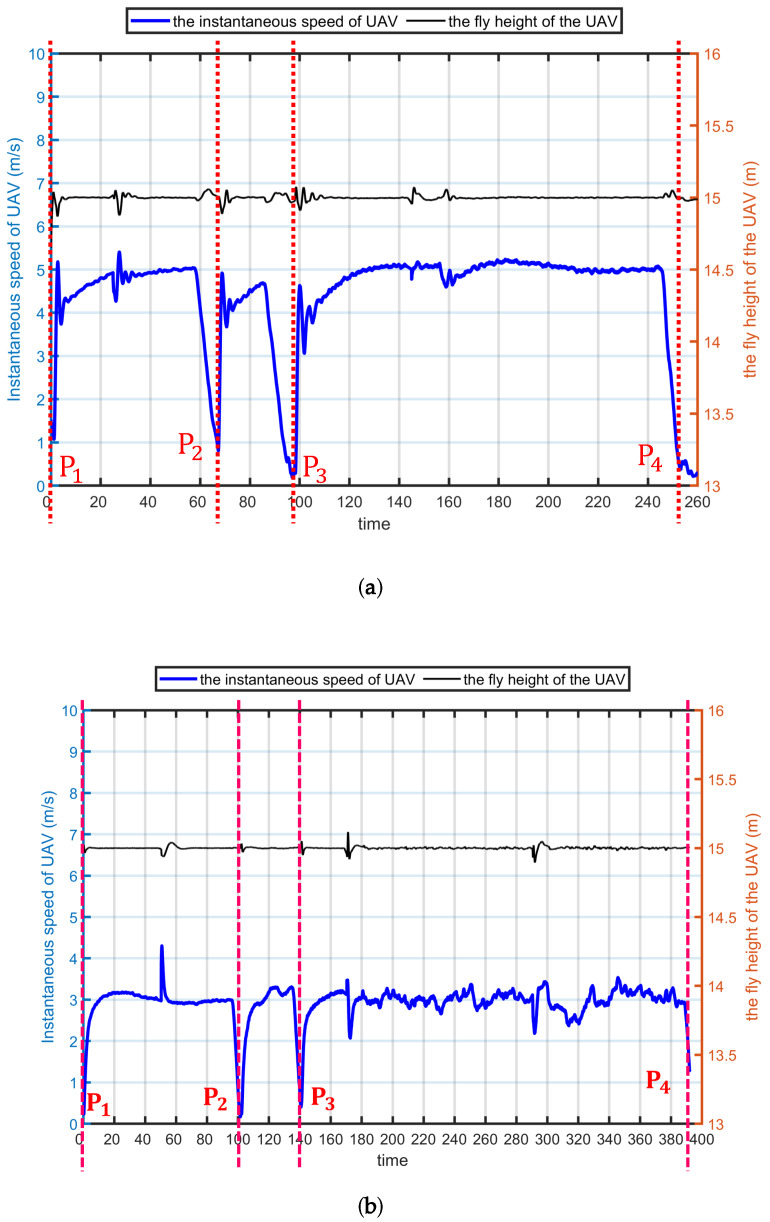
Presentation of the movements for UAV when it fly at 15 m with 3 ms^−1^ and 5 ms^−1^ in ground station. (**a**) The movements for UAV flying at 15 m, and its speed is 5 ms^−1^ in ground control station. (**b**) The movements for UAV flying at 15 m, and its speed is 3 ms^−1^ in ground control station.

**Figure 14:**
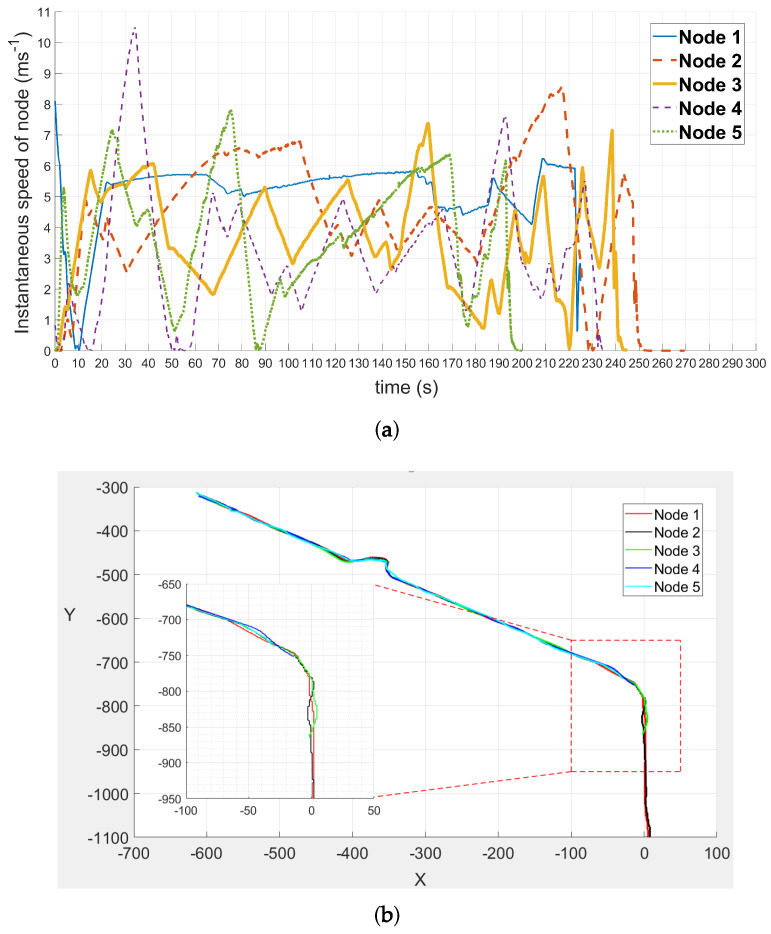
The movements for five nodes. (**a**) Instantaneous speeds of five nodes over time. (**b**) Trajectories of five nodes.

Figure 13 presents the movements for $UAV_{1}$ (only one UAV is used in real experiments) where the fly height is 15 m, with control speeds of 5 ms^−1^ (Figure 13a) and 3 ms^−1^ (Figure 13b) according to Pixhawk. In the studied experiments, the UAV flew at 15 m and 30 m. Figure 14 is an example to present the instantaneous speeds and trajectories for 5 nodes (Node 1 to Node 5) according to the Pixhawk.

To make the UAV fly along this path, we set four waypoints along the path as shown in Figure 12. In the experiments, the UAV start from $Point_{1}$ to achieve its given speed (it is 5 ms^−1^ in Figure 12) to $Point_{2}$, $Point_{3}$ and the ending point ($Point_{4}$). In Pixhawk autopilot system, the UAV will hover on the waypoint and ending point for 2 s. That is why in the Figure 13a, the UAV speed is lower than 5 ms^−1^ at $P_{2}$ and $P_{3}$. In Figure 13b, both the height and instantaneous speed of UAV have a shock between the $Point_{3}$ and $Point_{4}$ because of the influence of wind. The wind has an impact on the dynamic parameters so as to affect the relative velocity between the mobile node and the UAV, the network performance affected accordingly. However, it cannot be control during experiments.

#### 5.2.2. Results

**Figure 15:**
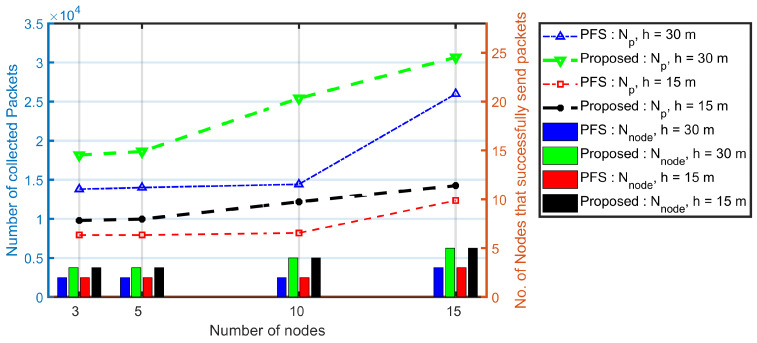
The impact of network size, and flying height over the system performance. In these experiments, the beacon interval is fixed at 2 s according to the simulation results in Figure 7.

**Figure 16:**
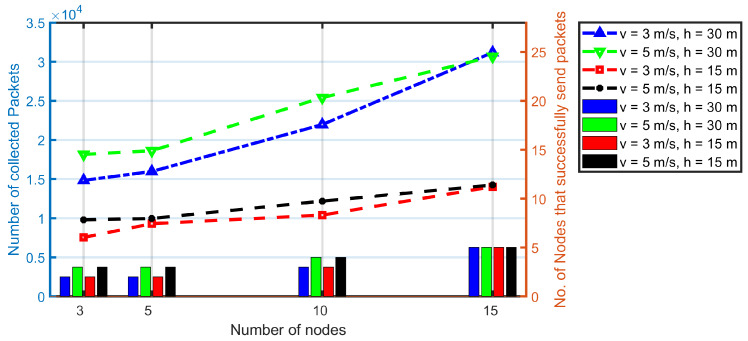
The impact of network size, and UAV’s speed over the system performance. In these experiments, the beacon interval is fixed at 2 s. All the results are based on the combination of Balance and PCdFS algorithms.

Figure 15 and Figure 16 show the experiments results under the proposed algorithms, the combination of the Balance algorithm and the PCdFS algorithm. From Figure 15, the number of collected packets in simulation is almost two times larger than in the experiments because of the impacts of hardware and environments are not considered in simulations. The flying height has a significant impact on the number of collected packets in experiments, especially when $N_{node}$ is steady between different heights. The higher the height, the larger number of nodes in both PFS and proposed algorithms. The number of collected packets of the proposed algorithm in size 15, h=30 m is more than twice than in h=15 m. The system performance increase as the size increase. The larger the network, the more nodes have opportunities to send packets, the more packets were collected. The number of collected packets in the proposed algorithm (when *h* is 15 m) is 1.2 times larger than in the PFS algorithm.

From Figure 16, it can be found that the UAV’s speed has little impact on data collection in real experiments. This is because the UAV’s speed is set at 3 ms^−1^ and 5 ms^−1^ because of the battery constrictions and the campus constrictions. The nodes’ speeds are between 2 ms^−1^ and 5 ms^−1^ also (Figure 14). Thus, the relative velocity between the UAV and mobile nodes is very small. The number of collected packets presented in Figure 16 keep the same conclusions as in simulations in Section 5.1.3 where the UAV fly at 5 ms^−1^ and 10 ms^−1^.

The flight height almost has no influence on the number of nodes that successfully transmit packets to the UAV, as presented in both Figure 15 and Figure 16, which are the same as in Section 5.1.3.

### 5.3. Discussions

According to the aforementioned simulations, the beacon interval and the UAV speed have a huge impact on network performance. The shorter the beacon interval, the better the system performance. The UAV speed is constrained by the node speed. The smaller the relative velocity between them, the higher the network performance. It keeps the same conclusions as in the real experiment. In real experiments, the data collection is well conducted when the UAV speed is set at 5 ms^−1^ which is very close to the average speed of mobile nodes. Compare to the other dynamic parameters, the number of priority levels has a steady impact on data collection in the simulations. From the movements of the nodes in Figure 14, it can be seen that the difference between the trajectories of nodes is very small because the road width is 5 m and the road length is 1200 m.

Compare Figure 13a,b, it also can be found that, the fly time in 3 ms^−1^ is 1.56 times as in 5 ms^−1^ while the speed increase by 66.67% (from 3 ms^−1^ to 5 ms^−1^). In the studied scenario, there are very small differences between the trajectories when UAV fly at 3 ms^−1^ and 5 ms^−1^ because the UAV follow the same path which width is very short compared to its length. Thus, the fly time is mainly dependent on the speed of the UAV. In other words, the slower the UAV fly, the higher energy consumption of the battery energy. From Figure 16, we notice that, the data collection has very little difference when UAV fly at 3 ms^−1^ and 5 ms^−1^. Therefore, under given constrictions, the higher the fly speed of the UAV, the more saved battery energy.

The fly height has very little impact on data collection in simulations because of the same transmission rate is adopted. However, the fly height has a huge impact on data collection in experiments because a real and complex antenna system are conducted among the transmissions between the node and the UAV. The higher the flying height, the less interference from external factors (e.g., buildings, etc.). Thus, the better the transmission, the higher the network performance.

## 6. Conclusions

In this paper, we developed two mechanisms: PCdFS and PMCdFS. PCdFS mechanism is used to build the scheduling communications when the nodes are only covered by one of the UAVs. PMCdFS is used to balance the communication between the nodes and multi-UAVs when these nodes within the range of multi-UAVs at the same time. Based on the two mechanisms, we proposed the Balance algorithm which highly enhances the network fairness in the applications where both the nodes and the collectors are mobile. Two key mechanisms for designing Balance algorithm are: (i) divide the interesting areas into different priority areas and (ii) provide an independent priority value for each node in the same priority group according to their contact duration with the UAVs. We examined the performance of proposed algorithms through extensive simulations, and real experiments. In the experiments, we used 15 mobile nodes at a path with several intersections and one island at the Tongji campus in Shanghai, China. We also confirm the applicability of the proposed algorithm in a challenging and realistic scenario through numerous experiments. Both simulation results and experiment results present that the proposed PCdFS algorithm enhanced the network performance efficiently. The backhaul dimensioning is an interesting problem that we will address in our future work. It depends on the used backhaul type (either satellite or terrestrial) and on the allocation that is reserved to the network slice dedicated for Machine Type Communication (MTC) traffic.

## Figures and Tables

**Table 1:** Summary of related works.

Ref.	Sensor Status	$N_{uav}$	Descriptions
[6]	Static deployed	1	Through UAV trajectory planning to achieve timely data collection from IoT devices where the data has deadlines and needs to be sent before the data loses its meaning or becomes irrelevant.
[7]	Static deployed	1	Considering the age of information, characterized by the data uploading time and the time elapsed since the UAV leaves a node, when designing the UAV trajectory.
[11]	Static deployed	1	To extend the lifetime of the network through charging for the UAV in the air.
[14,15]	Static deployed	1	The authors divided the interesting area into different priority groups, and the data communication conducted from higher to lower priorities (PFS mechanism). Based on PFS, the authors proposed MAC protocols for UAV-aided WSN.
[24]	Static deployed	1	the authors through optimizing the trajectory of a rotary-wing UAV to collect data with an objective of minimizing the maximum energy consumption of all devices.
[25]	Static deployed	1	To minimize the flight time, and jointly optimize the transmit power of nodes, the UAV speed and the transmission intervals.
[31]	Static deployed	1	To minimize the energy consumption of the system through optimizing the UAV’s trajectory and devices’ transmission schedule, while ensuring the reliability of data collection and required 3D positioning performance.
[32]	Static deployed	1	To maximize the minimum average data collection rate from all nodes subject to a prescribed reliability constraint for each node by jointly optimizing the UAV communication scheduling and three-dimensional trajectory.
[33]	Static deployed	1	To minimize the maximum delay of all ground users through jointly optimizing the offloading ratio, the users’ scheduling variables, and UAV’s trajectory.
[34]	Static deployed	1	To maximize the minimum received energy of ground users by optimizing the trajectory of the UAV. They first presented the globally optimal one-dimensional (1D) trajectory solution to the minimum received energy maximization problem.
[20]	Static deployed	Multiple	Minimize the maximum mission completion time through jointly optimize the wake-up scheduling and association for sensors, the UAV trajectory, while ensuring that each node can successfully upload the targeting amount of data with a given energy budget.
[35]	Static deployed	Multiple	To maximize the data collection utility by jointly optimizing the communication scheduling and trajectory for all UAVs.
[36]	Static deployed	Multiple	The authors proposed a risk-aware trajectory planning algorithm for multi-UAVs for urban applications.
[37]	Static deployed	Multiple	To minimize the mission completion time of the UAVs through designing the UAV’s trajectory, and meanwhile, they guaranteed that each ground user can successfully recover the file.
[38]	Static deployed	Multiple	To maximize the minimum throughput of ground users through optimizing the trajectory for each UAV.
[39]	Static deployed	Multiple	To minimize the mission time by planning the trajectory of each UAV, while satisfying the time requirements.
[40]	Static deployed	Multiple	Use nested Markov chains to analyze the probability for successful data transmission, and propose a sense-and-send mechanism for real-time sensing missions, and a multi-UAVs based Q-learning algorithm for decentralized UAV trajectory planning.
this paper	Mobile	Multiple	Collect data from mobile nodes through balancing the different contact durations between mobile nodes, and multi-UAVs.

**Table 2:** Major notations used in this article.

Parameters	Descriptions
*N*	Network size;
*M*	The number of UAVs;
$UAV_{i}$	The *i*th UAV;
S	The sensors set;
$S_{kB}^{i}$	The sensors set that within the range of $UAV_{i}$ in $t_{k}$;
$S_{kB}^{i,o}$	The sensors set that only within the range of $UAV_{i}$ in $t_{k}$;
$S_{kB}^{i,j}$	The sensors set that within the range of both $UAV_{i}$ and $UAV_{j}$ in $t_{k}$;
U	The UAVs set;
$T_{i}$	The collection period of the $UAV_{i}$;
$h_{i}$	The fly height of the $UAV_{i}$;
*L*	The path length;
$L_{i}$	The length of the trajectory of $UAV_{i}$;
$N_{ts}$	The number of time slots;
T	The set of time-slots;
α	The duration of one time-slot;
$P_{m}^{i}$,$P_{S}^{i}$,$P_{E}^{i}$	The “*m*th”, the “start”, and the “end” way points of the $UAV_{i}$ respectively;
$P_{i}$	The set of waypoints for $UAV_{i}$;
$\lambda_{m,n}^{i}$	The UAV’s flight time between any two waypoints $P_{m}^{i}$ and $P_{n}^{i}$ of $UAV_{i}$;
F	The set of nodes that send at least one packet in collection period;
$t_{B}$	The duration between adjacent two “Beacon”;
$N_{ts,a}\left(i,j,k\right)$	A matrix where value is “0” and “1”. $N_{ts,a}\left(i,j,k\right)=1$ implies in $t_{k}$, the $UAV_{i}$ will communicate with $S_{j}$, and othwise it is “0”;
$\sigma_{ijk}$	Boolean function. $\sigma_{ijk}=1$ implies that the $UAV_{i}$ successfully collect data from $S_{j}$ in $t_{k}$;
$N_{t,a}^{i}$	The number of time slots that sensor $S_{i}$ ($S_{i}\in S$) was allocated in time *T*;
$N_{p}$	The total number of collected packets;
$N_{node}$	The number of nodes that successfully send at least one packets.

**Table 3:** Proposed algorithms.

Algorithms	Descriptions
Balance mechanism	It is specially used to balance the communications between multiple nodes and multiple UAVs for the system.
PCdFS mechanism	It is used to build the “scheduling” between the nodes (that only with the range of a single UAV) and the UAV.
PMCdFS mechanism	It is specially used to balance the communications between the nodes (these nodes are within the range of multiple UAVs at the same time) and the UAV.

**Table 4:** Simulation parameters.

Parameter	Value	Parameter	Value
network size	[5, 200]	path width	10 m
fly time	5 min	fly height	[5, 95] m
UAV speed	[5, 25] ms^−1^	sensor speeds	[0, 10] ms^−1^
# priority groups	[2, 5]	packet size	127 Bytes
Data bit rate	250 kbps	inter-beacon duration	2 s to 60 s
receiving threshold	−70 dBm	sensing threshold	−80 dBm
transmission range of the UAV and the node		100 m	

**Table 5:** Summary of simulations.

Section	Parameters	$N_{\mathrm{uav}}$	Descriptions
Section 5.1.1. Impact of priority level changes.	*N* = 200, *h* = 15 m, *v* = 10 ms^−1^, IBD = 2 s, $v_{i}\in$ [0, 10] ms^−1^, $N_{pl}\in$ = {2,3,4,5}.	1	Study the impact of priority levels on the network performance.
Section 5.1.2. Varying beacon intervals.	*N* = 200, h∈ [5, 95] m, v∈ [5, 25] ms^−1^, IBD ∈ [2, 60] s, $v_{i}\in$ [0, 10] ms^−1^, $N_{pl}$ = 2.	1	Study the impact of different synchronization frequency on the network performance.
Section 5.1.3. Impact of UAV’s parameters changes.	*N* = 200, *h* = 15 m, *v* = 10 ms^−1^, IBD = 2 s, $v_{i}\in$ [0, 10] ms^−1^, $N_{pl}$ = 2.	1	Study the impact of fly height and speeds on the network performance.
Section 5.1.4. Scalability.	N∈ [5, 200], *h* = 15 m, *v* = 10 ms^−1^, IBD = 2 s, $v_{i}\in$ [0, 10] ms^−1^, $N_{pl}$ = 2.	1	Study the impact of the network size on the network performance.
Section 5.1.5. Comparison between Multi-UAVs and Single-UAV.	*N* = 200, *h* = 15 m, *v* = 10 ms^−1^, IBD = 2 *s*, $v_{i}\in$ [0, 10] ms^−1^, $N_{pl}$ = 2.	{1,2}	Compare our proposed algorithms when using one UAV and two UAVs.
